# Immune response to influenza vaccination in the elderly is altered by chronic medication use

**DOI:** 10.1186/s12979-018-0124-9

**Published:** 2018-08-31

**Authors:** Divyansh Agarwal, Kenneth E. Schmader, Andrew V. Kossenkov, Susan Doyle, Raj Kurupati, Hildegund C. J. Ertl

**Affiliations:** 1 Genomics and Computational Biology, Perelman School of Medicine, University of Pennsylvania, Philadelphia, PA 19104 USA; 2 Division of Geriatrics, Duke University Medical Center; Geriatric Research, Education, and Clinical Center, Durham VA Medical Center, Durham, NC 27705 USA; 3 The Wistar Institute, Philadelphia, PA 19104 USA

**Keywords:** Influenza vaccine, Metformin, NSAIDs, Statin, Immune response

## Abstract

**Background:**

The elderly patient population is the most susceptible to influenza virus infection and its associated complications. Polypharmacy is common in the aged, who often have multiple co-morbidities. Previous studies have demonstrated that commonly used prescription drugs can have extensive impact on immune defenses and responses to vaccination. In this study, we examined how the dynamics of immune responses to the two influenza A virus strains of the trivalent inactivated influenza vaccine (TIV) can be affected by patient’s history of using the prescription drugs Metformin, NSAIDs or Statins.

**Results:**

We provide evidence for differential antibody (Ab) production, B-cell phenotypic changes, alteration in immune cell proportions and transcriptome-wide perturbation in individuals with a history of long-term medication use, compared with non-users. We noted a diminished response to TIV in the elderly on Metformin, whereas those on NSAIDs or Statins had higher baseline responses but reduced relative increases in virus-neutralizing Abs (VNAs) or Abs detected by an enzyme-linked immunosorbent assay (ELISA) following vaccination.

**Conclusion:**

Collectively, our findings suggest novel pathways that might underlie how long-term medication use impacts immune response to influenza vaccination in the elderly. They provide a strong rationale for targeting the medication-immunity interaction in the aged population to improve vaccination responses.

**Electronic supplementary material:**

The online version of this article (10.1186/s12979-018-0124-9) contains supplementary material, which is available to authorized users.

## Background

The trivalent inactivated influenza vaccine (TIV) protects against severe influenza by inducing the production of virus neutralizing Abs. According to recent estimates by the Center for Disease Control & Prevention (CDC), between October 1, 2017 and February 3, 2018, clinical laboratories tested more than 600,000 specimens for influenza virus, approximately 125,000 of which tested positive [1]. Among individuals who tested positive, the majority had influenza A, subtypes H3N2 and H1N1. More than a third of these cases were patients ≥65 years old, who experienced more severe illness than other age groups. Not only did they account for more than half of the influenza-associated hospitalizations, their mortality rates were also highest.

Recent studies have demonstrated that in people over 65 years of age, the vaccine efficacy against influenza A viruses is less than 20% [2, 3]. Vaccine effectiveness in the elderly is variable, secondary to a variety of reasons such as ethnic background, inflammatory responses at baseline, timing of vaccination, gender and prior vaccination history [2–5]. Furthermore, primary B-cell responses in the elderly are commonly low and short-lived, resulting in Abs with low affinity [6]. A decrease in germinal center formation and decline of the B and T lymphocyte attenuator (BTLA) expression on B cells during immunosenescence also contribute to lack of sustained Ab responses in the aged [7]. Imperfectly matching antibodies due to antigenic sin may further result in poor responses to the influenza vaccine and suboptimal pathogen control in the aged population. The ‘original antigenic sin’ concept refers to the impact of the first encounter with antigens of an influenza virus on lifelong immunity [8]. When a virus strain undergoes antigenic drift, some epitopes remain conserved, and pre-existing antibodies to such epitopes cross-react to the drifted strain. This in turn suppresses antigen levels through epitope masking and Fc-mediated mechanisms. Consequently, pre-existing influenza virus-specific antibody responses are boosted while the diversity of the overall response is diminished. This becomes increasingly pronounced in the aged who are typically exposed multiple times to different influence A virus variants. Additionally, it has been shown that the defects in B-cell responses in the elderly are related to a decline of helper functions from CD4^+^ T-cells [9], and reduced expression of costimulatory receptors, such as CD28 and CD40L, which are essential for B-cell activation and germinal center formation [10].

Although our understanding of the complex biological processes that regulate vaccine responses in the elderly has evolved over the past decade, it is yet unclear how long-term medication use in individuals with chronic diseases affects immune responses to vaccination. While some vaccine–drug interactions, such as influenza–warfarin, have been reported, it is likely that many others remain unrecognized. A non-specific mechanism underlying the interaction between the influenza vaccine and medicines such as warfarin, phenytoin and theophylline is suggested to be due to the vaccine’s ability to inactivate the hepatic cytochrome P-450 system, thereby reducing drug clearance [11]. More recently, studies have found that virus neutralizing Ab (VNA) titers to influenza A (H1N1 and H3N2), and B strains were lower in subjects receiving chronic Statin therapy, compared with those not receiving this drug and that vaccine effectiveness against the H1N1 strain was reduced [12, 13] . Relatedly, it has been shown that a Type 2 Diabetes (T2D) medication, Metformin, increases the proportion of circulating switched memory B-cells, decreases the fraction of exhausted memory B-cells, and reduces B-cell-intrinsic inflammation [14]. Consequently, higher VNA titers in response to influenza vaccination have been associated with Metformin use in patients [14]. These observations have underscored the importance of examining the effects of chronic drug use on vaccine responses in order to both better understand how the drugs alter immunity in the aged, as well as design supplementary therapeutic approaches for the elderly who are vulnerable to influenza virus infections, and comprise the largest customer segment for medications prescribed for chronic conditions.

We sought to examine this question in a 5-year study of TIV responses in the healthy, community-dwelling elderly (≥65 years of age) by (i) collecting prior drug-use data on each patient, (ii) testing each individual’s Ab responses to the two influenza A viruses of the vaccine, H1N1 and H3N2, (iii) measuring proportions and phenotypes of different B-cell subsets, and (iv) performing genome-wide gene expression analysis of whole-blood. These immunological and clinical parameters were recorded at baseline (pre-vaccination) and on days 7 and 14 or 28 after vaccination, which allowed us to examine longitudinal trends. The overall aim of our study was to analyze how chronic drug use of commonly prescribed medicines in the elderly might affect responses to the influenza vaccine. We wanted to correlate post-vaccination changes in the immune system with the patient’s prior history of drug use. These associations would allow us to assess the impact of polypharmacy on how the aging human body responds to the influenza vaccine. By providing a more complete picture of how the medication–immunity interaction changes vaccination responses, this study may help guide the development of alternative strategies to promote long-lasting immunity and better protect the at-risk, aging population.

## Results

### Patient characteristics

Between 2011 and 2016, we recorded 164 vaccinations in 80 individuals. Of these, data for thirteen patients was filtered out upon initial quality control because either they had missing values for Ab measurements or several B-cell phenotypic parameters, or we were unable to obtain reliable micro-array data on their transcriptome. Thus, for subsequent analyses, we examined data from patients for whom complete datasets were available, which included 131 vaccinations in 67 individuals with a mean age of 76.3 years (Table 1: Summary of Patient Characteristics). Some individuals were enrolled repeatedly during the 5-year study period. Each vaccination was considered as independent and identically distributed (*iid*) data, an assumption necessary to conduct statistical inference on the dataset. We defined the “elderly” population as individuals ≥65 years of age. The maximum age in our cohort was 89 years. The majority of the human subjects in our study were White/Caucasians (91.6%), followed by African-Americans (5.3%) and others (3.1%). We recorded prior clinical history of drug use for each patient, and converted this information to a binary variable: 1, if the patient had one year or greater of medication use and was using the drug at the time of assessment, and 0 otherwise. We divided the patient cohort into two categories for each of the three drugs — Metformin, Non-Steroidal Anti-inflammatory Drugs (NSAIDs) and Statins. Fifty-two percent of our observations corresponded to chronic NSAIDs users. In contrast, only 32% and 8% of the enrolled elderly used Statin and Metformin, respectively. Females comprised most of the subjects in our study, 65.6%. Sixty-five percent of the vaccinations were also performed before noon and the others were done in the afternoon.

**Table 1:** Summary of Patient Characteristics

Mean Age		76.3 years
	# Visits	%
Total	131	
Female	86	65.6
Male	45	34.4
Vaccinated in AM	85	64.9
Vaccinated in PM	46	35.1
Race		
Caucasian	120	91.6
Black	7	5.3
Others	4	3.1
Metformin-Users	11	8.4
Female	8	72.7
Male	3	27.3
NSAIDS-Users	68	51.9
Female	47	69.1
Male	21	30.9
Statin-Users	42	32.1
Female	22	52.4
Male	20	47.6

### VNA titer and Ig ab levels provide evidence for drug–immunity interaction

**Fig. 1:**
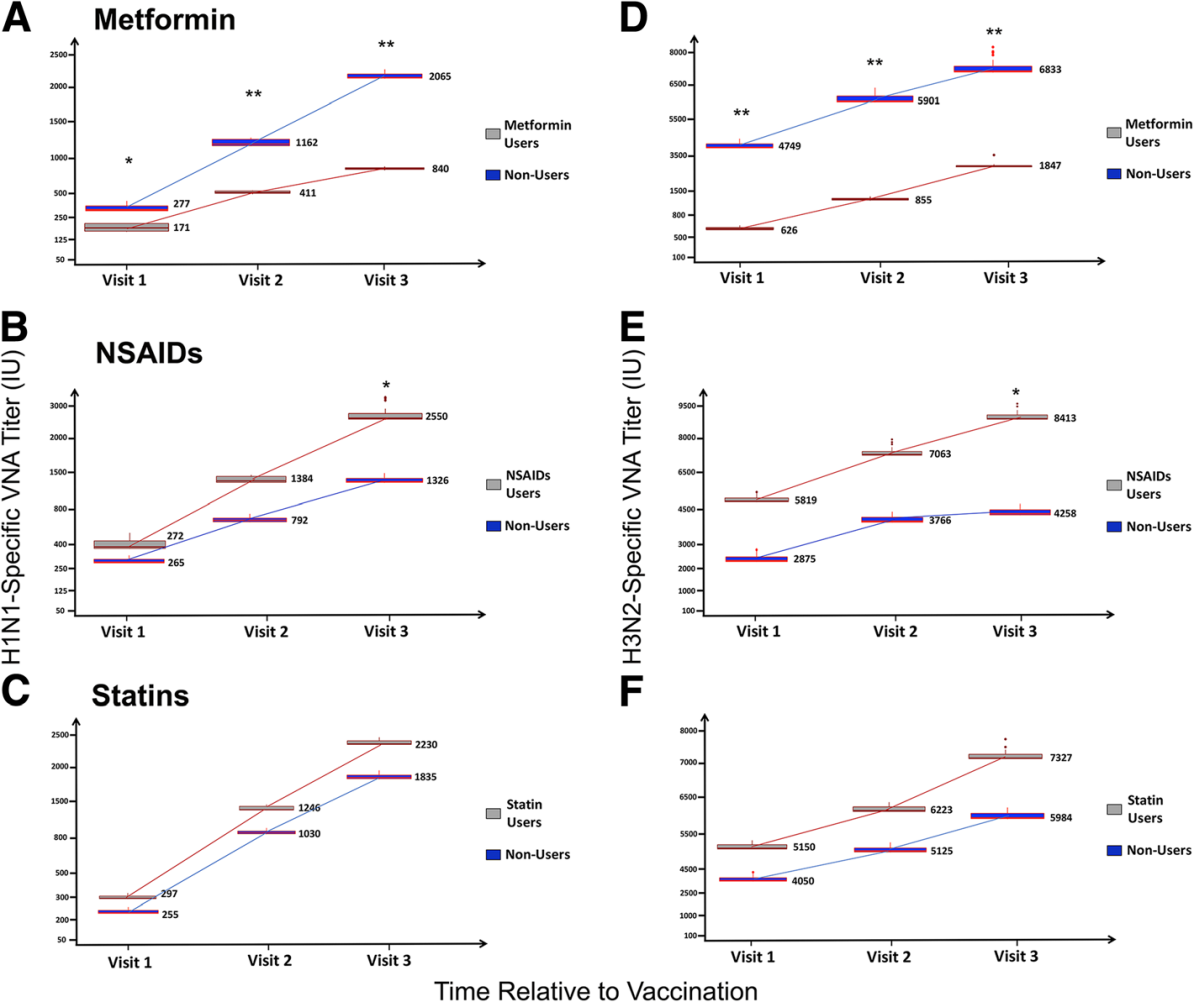
Influenza virus-specific VNA titers: Virus neutralizing antibody (VNA) responses are summarized between drug-users and non-users for each of the three drug categories examined**.** The line within the box plots show medians (the values are written adjacent to the boxes), and the boxes show the interquartile range for the titer levels of H1N1- (**a**-**c**) and H3N2-specific (**d**-**f**) VNAs in sera. Top panels **a**, **d** show VNA titers for 11 samples from individuals on chronic Metformin use compared with 120 samples from non-users. Middle panels **b**, **e** show the titers for 68 and 63 samples, respectively, from individuals with or without a history of NSAIDs use and the bottom panels **c**, **f** show the titers for 42 samples from Statin users, compared with 89 samples from non-users. Pair-wise differences between the two cohorts in each of the six panels were performed using the non-parametric Mann-Whitney U Test. Stars above boxes indicate significant differences * *p*-value ≤0.05, ** *p*-value ≤0.005

We determined the VNA titers and quantified immunoglobulin (Ig) isotypes to H1N1 and H3N2 at baseline (Day 0, visit 1), and at one (visit 2) and two or four weeks (visit 3) after vaccination. Based on a comparison of absolute values, we found that at all three time points, Metformin users had significantly lower VNA titer to H1N1 compared with non-users. This difference became more pronounced after vaccination, when by visit 3 Metformin users were found to have significantly lower titers (840 international units [IU] versus 2065 IU in non-users (Fig. 1a), *p*-value < 0.05). When the visit 1 and 2 titer levels were normalized by baseline levels, the poor responses in Metformin users became more apparent – at visit 2, they witnessed a 3-fold increase in H1N1 VNA titers, compared with a 20-fold increase in non-users (*p*-value =0.002), and at visit 3, the latter cohort experienced a 37-fold increase in titers compared with a mere 5-fold increase in the Metformin users (*p* = 0.003). Similarly, we found that Metformin users tend to have lower H3N2 VNA titers at all time points of the study. At baseline, the users had a mean titer of 625 IU compared with 4749 IU in non-users (*p*-value =2 × 10^− 4^). This significant difference was maintained with samples collected at visits 2 and 3 (Fig. 1d).

NSAIDs users had higher VNA titers to H1N1 and H3N2 at baseline, although increases in titers were comparable between users and non-users. (Fig. 1b, e). At subsequent visits 2 and 3, NSAIDs users had higher absolute titers compared with non-users. Individuals on Statin therapy also tended to have higher baseline VNA titers compared with non-users although this difference did not reach the threshold for statistical significance. Interestingly, the degree of fold-change in titers was similar between the Statin users and non-users (Fig. 1c, f).

**Fig. 2:**
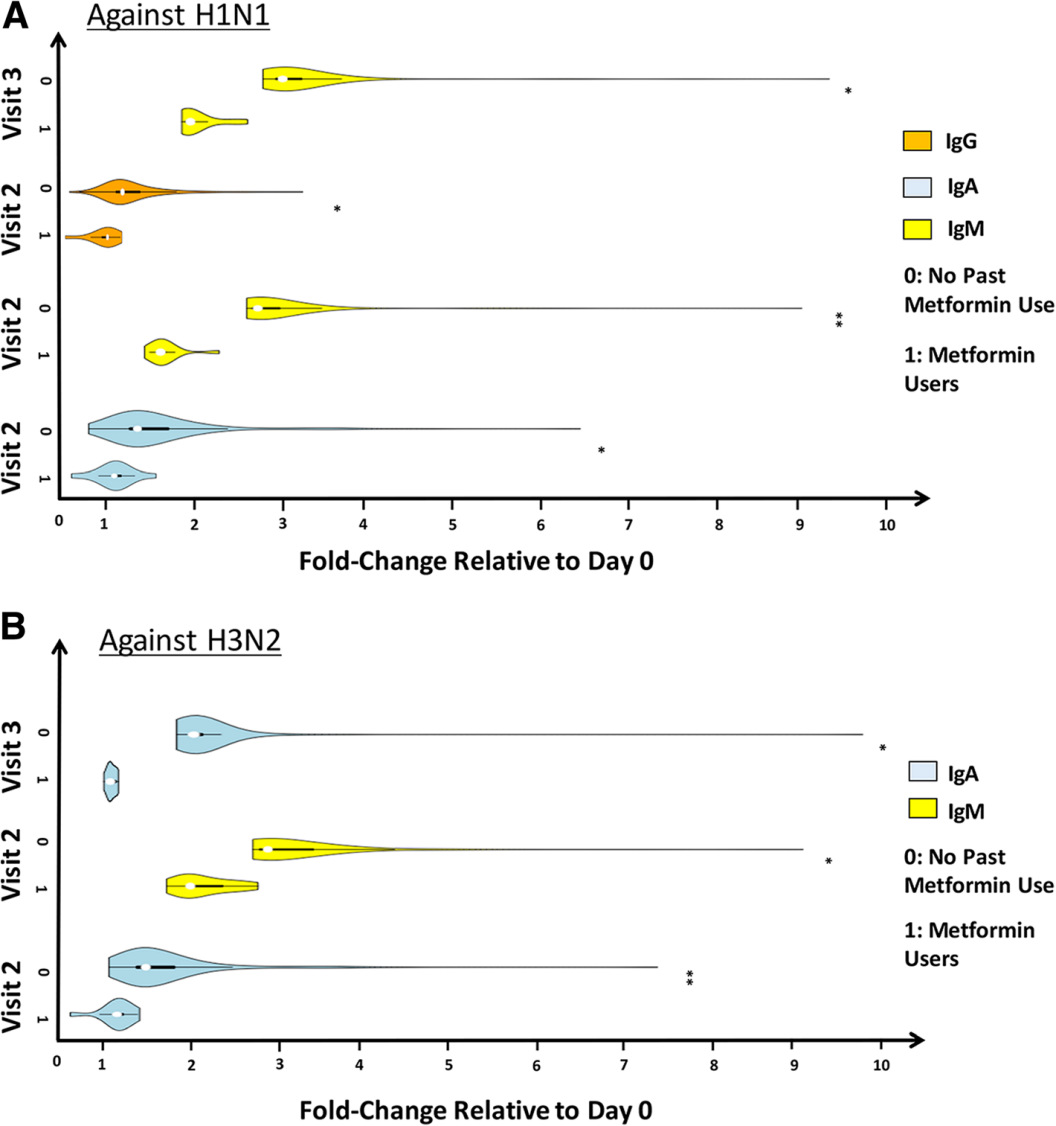
Isotypes of influenza virus-specific antibodies. Antibody isotype responses to H1N1 and H3N2 viruses in elderly individuals with a history of Metformin use compared to individuals with no prior drug use history are shown. Serum samples collected before and on two separate visits following vaccination were tested for IgA, IgG and IgM to H1N1 (**a**) and H3N2 (**b**) viruses by ELISA. The violin plots show the median Ab increases relative to pre-vaccination titers in elderly individuals with a history of Metformin use (1 on the Y-axis) compared with individuals with no prior drug use history (0 on the Y-axis). The white dots in within the plots show the median results, the bold black line within the plots shows the interquartile range, the thickness of the plot indicates the distribution of changes. Pair-wise differences between the two cohorts in each of the six panels were performed using the non-parametric Mann-Whitney U Test. Stars on the right of the plots indicate significant differences: * a *p*-value ≤0.05, ** *p*-value ≤0.005

In addition to VNA titer differences, we observed that individuals on long-term Metformin therapy had significantly lower IgA, IgG and IgM responses to the H1N1 strain after vaccination. Compared with non-users, they exhibited a diminished IgM Ab response up to visit 3 (Fig. 2a). Similarly, we observed diminished IgA and IgM Ab production against the H3N2 strain in Metformin users at visit 2. After an additional week, the IgA Ab levels in Metformin users were significantly lower compared with non-users (Fig. 2b; *p*-value < 0.05). No significant differences in Ig-Ab responses were noticeable in the NSAIDs cohort (data not shown). Individuals with a history of long-term Statin use displayed reduced IgG and IgM levels against both strains of the influenza vaccine. They had significantly lower IgG levels to the H3N2 strain, and considerably lower IgM and IgG levels against the H1N1 strain at visits 2 and 3 (see Additional file 1: Figure S1).

### B-cell phenotypic markers provide insight into the effects of chronic medication use on vaccine response

Encouraged by our preliminary results of differential responses to the influenza vaccine conditional on the medication use history among the elderly, we sought to examine the underlying mechanisms, which might explain those differences. Using flow cytometry, we defined five subsets of B-cells, viz., transitional, mature naïve, non-switched memory, switched memory and Ab secreting cells (ASCs), and then tested for the expression of B-cell phenotypic or metabolic markers. B and T lymphocyte attenuator (BTLA), Programmed cell death protein (PD) 1, Programmed death-ligand (PD-L) 1 and 2, mitochondrial reactive oxygen species (ROS), B-cell lymphoma (Bcl)-6, Octamer transcription factor (Oct)-2 and Sirtuin (Sirt) 1 showed differences in mean florescence intensity (MFI) levels in drug users. These markers were chosen based on their role in regulating different physiological aspects of B-cell differentiation and metabolism. BTLA and PD1 are immunoinhibitors, which interact with the herpes virus entry mediator or PD-L1/L2 respectively [15, 16]. They also play a role in interactions between B-cells and follicular T-helper cells. Bcl-6 plays a pivotal role in germinal center maturation of B-cells [17], while the transcription factor Oct-2 sponsors differentiation of B-cells into Ab secreting plasma cells [18]. As B-cell differentiation is also driven by metabolic cues we tested for a number of markers indicative of pathways of energy production. An increase in ROS is observed in cells that enhance energy production through mitochondrial oxidative phosphorylation [19]. Sirt1, a nuclear energy sensor that becomes activated by NAD^+^, increases fatty acid oxidation and gluconeogenesis [20]. Sirt1 reduces inflammatory reactions through inhibition of NF-κB, enhances insulin secretion and glucose tolerance and regulates circadian rhythm [20, 21]. Sirt1 also inhibits regulatory T (T_reg_) cell formation, and enhances Th17 cell generation [22]. In B-cells, it promotes cytokine production [23]. Its activity also increases upon caloric restriction as well as in response to DNA damage that occurs during B-cell class switching reactions. Depending on the type of DNA damage, Sirt1 promotes cell survival and affects overall lifespan [24, 25].

**Fig. 3:**
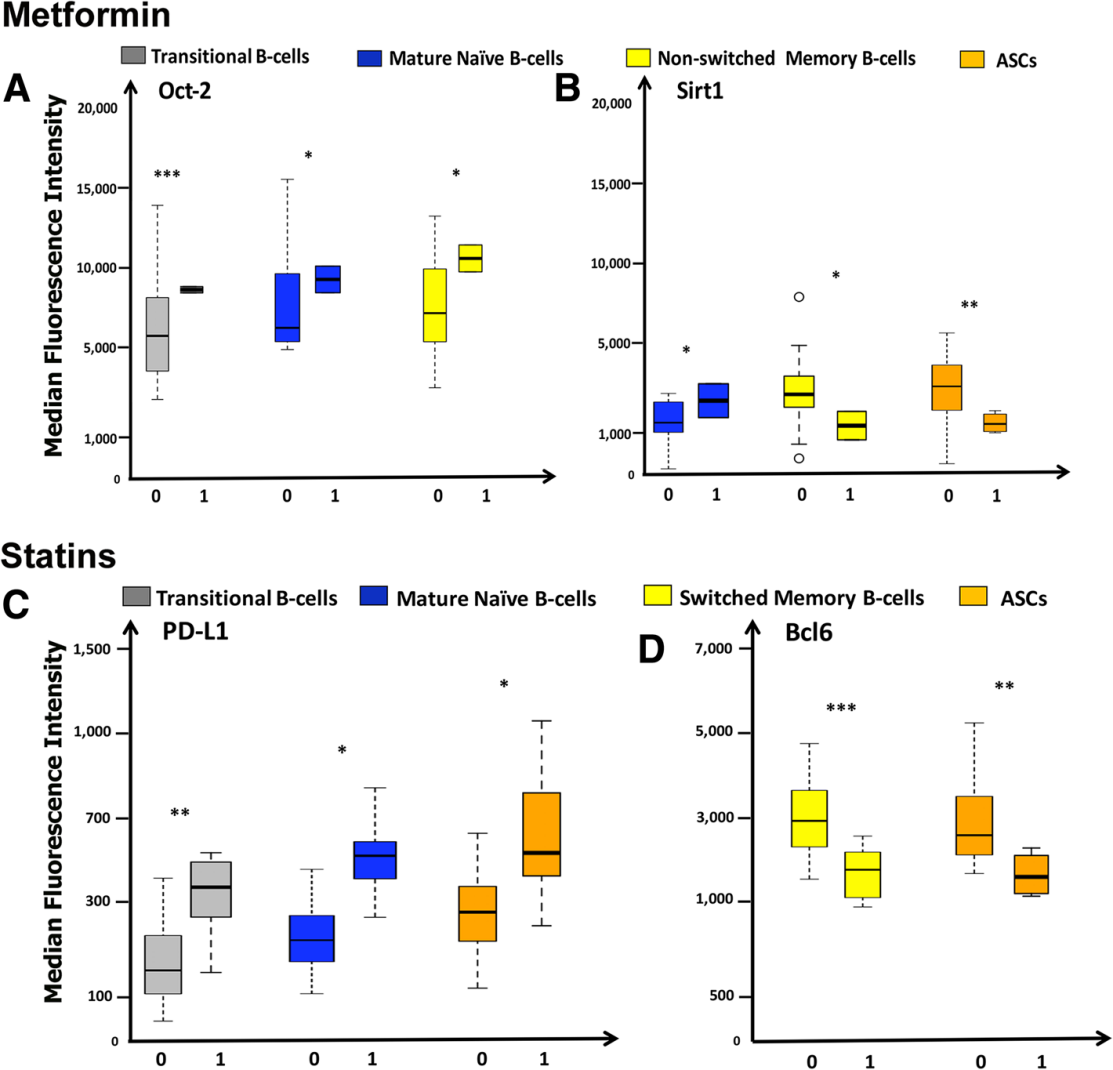
Phenotypes of B cell subsets. The boxplots show MFI levels of significantly altered markers on different B-cell subsets (using flow cytometry) from individuals with no history of medication use (0 on the X-axis), compared with individuals using Metformin or Statins (1 on the X-axis). The line within the box plots show medians, the boxes show the interquartile range and the lines show maximal and minimal data. Panels **a** and **b** display elevated levels of Oct-2 and Sirt1, respectively, in various B-cell subsets in Metformin users. Panels **c** and **d** demonstrate elevated levels of PD-L1 and decreased levels of Bcl6, respectively, in various B-cell subsets in Statin users. Pair-wise differences between the cohorts in each panel were performed using the Mann-Whitney U Test. Stars above the boxes indicate significant differences: * *p*-value ≤0.05, ** *p*-value ≤5 × 10^− 3^ and ***p-value ≤5 × 10^− 4^

We found numerous B-cell markers that were significantly altered in the cohort of individuals using either of the mediations under study, when compared with their corresponding non-drug user cohorts using two-sample t-tests. Among the Metformin users, we found significantly elevated MFI levels of Oct-2 in transitional, mature naïve and non-switched memory B-cells (Fig. 3a). We also found elevated levels of Sirt1 in mature naïve B-cells, although non-switched memory B-cells and ASCs in Metformin users had decreased levels of Sirt1 compared to non-users (Fig. 3b). Additionally, we detected elevated levels of BTLA and PD1 in the mature naïve B-cells among the Metformin users (see Additional file 2: Figure S2).

Among the individuals who were long-term users of NSAIDs, the switched memory B-cell subset was found to have significantly higher MFI levels of mitochondrial reactive oxygen species (MROS) and diminished levels of Bcl-6 (see Additional file 3: Figure S3). Similarly, the mature naïve B-cells in the NSAIDs user group exhibited dramatically greater intensity of BTLA and PD1 (see Additional file 2: Figure S2); *p*-value < 0.005, for each pair-wise comparison). When examining the individuals on long-term Statin therapy, levels of PD-L1 were found to be raised in transitional B-cells, mature naïve B-cells and ASCs (Fig. 3c). ASCs and switched memory B-cells in Statin users were also observed to have much lower MFI of Bcl-6 compared with non-users (Fig. 3d).

### Whole-transcriptome analysis and pathway-level alterations

Whole blood gene expression profiles obtained at baseline and at visit 2 after vaccination showed differential expression of 51 genes (FDR < 10%) in individuals receiving Metformin compared to the non-users; the majority of these genes were downregulated in the Metformin-user group and coded for extracellular exosomal proteins, as well as enzymes involved in protein digestion, histamine biosynthesis and the LPS/IL-1-mediated inhibition of RXR function ((see Additional file 4: Figure S4); Table 2).

**Table 2:** Signature Pathways Altered Between the Drug-User and Non-User Cohorts based on the significant DE genes. Pathway enrichments were assessed using the Laboratory of Immunopathogenesis and Bioinformatics resource, DAVID. Only Pathway associations with a *p*-value < 0.05 and an FDR < 10% were considered statistically noteworthy

Top Canonical Pathways associated with DE genes	FDR (%)	*p*-value
METFORMIN		
Extracellular Exosome	6.2	2.5 × 10–3
LPS/IL-1 Mediated Inhibition of RXR Function	8.1	1.1 × 10–3
Histamine Biosynthesis	8.1	2.0 × 10–3
Protein Digestion	8.5	2.8 × 10–4
NSAIDS		
Antiviral Defense	2 × 10–2	5.6 × 10–7
Cell Division and Chromosome Partitioning	9.8 × 10–2	6.6 × 10–6
type I interferon signaling pathway	0.16	3.2 × 10–6
Inhibition of Viral Genome Replication	2.2	3.5 × 10–5
Innate Immunity	5.7	4.7 × 10–4
STATIN		
Antigen Presentation Pathway	1 × 10–3	1.5 × 10–7
Interferon-gamma-mediated signaling pathway	1.9 × 10–3	2.9 × 10–8
Regulation of interleukin-10 secretion	5.1 × 10–3	2 × 10–9
OX40 Signaling Pathway	8.7 × 10–3	5.2 × 10–6
Regulation of interleukin-4 production	1 × 10–2	7.8 × 10–9
B Cell Development	1.5 × 10–2	1.2 × 10–5
Cdc42 Signaling	3.4 × 10–2	5.7 × 10–5
Nur77 Signaling in T Lymphocytes	3.4 × 10–2	5.7 × 10–5
Th1 and Th2 Activation Pathway	4.3 × 10–2	8.7 × 10–5
Dendritic Cell Maturation	4.6 × 10–2	1 × 10–4
iCOS-iCOSL Signaling in T Helper Cells	0.17	5.1 × 10–4
CD28 Signaling in T Helper Cells	0.19	6.3 × 10–4
Phagosome Maturation	0.26	8.7 × 10–4
PKCtheta Signaling in T Lymphocytes	0.3	1.1 × 10–3
Role of NFAT in Regulation of the Immune Response	0.46	1.7 × 10–3
Role of JAK family kinases in IL-6-type Cytokine Signaling	7.1	3.1 × 10–2

**Fig. 4:**
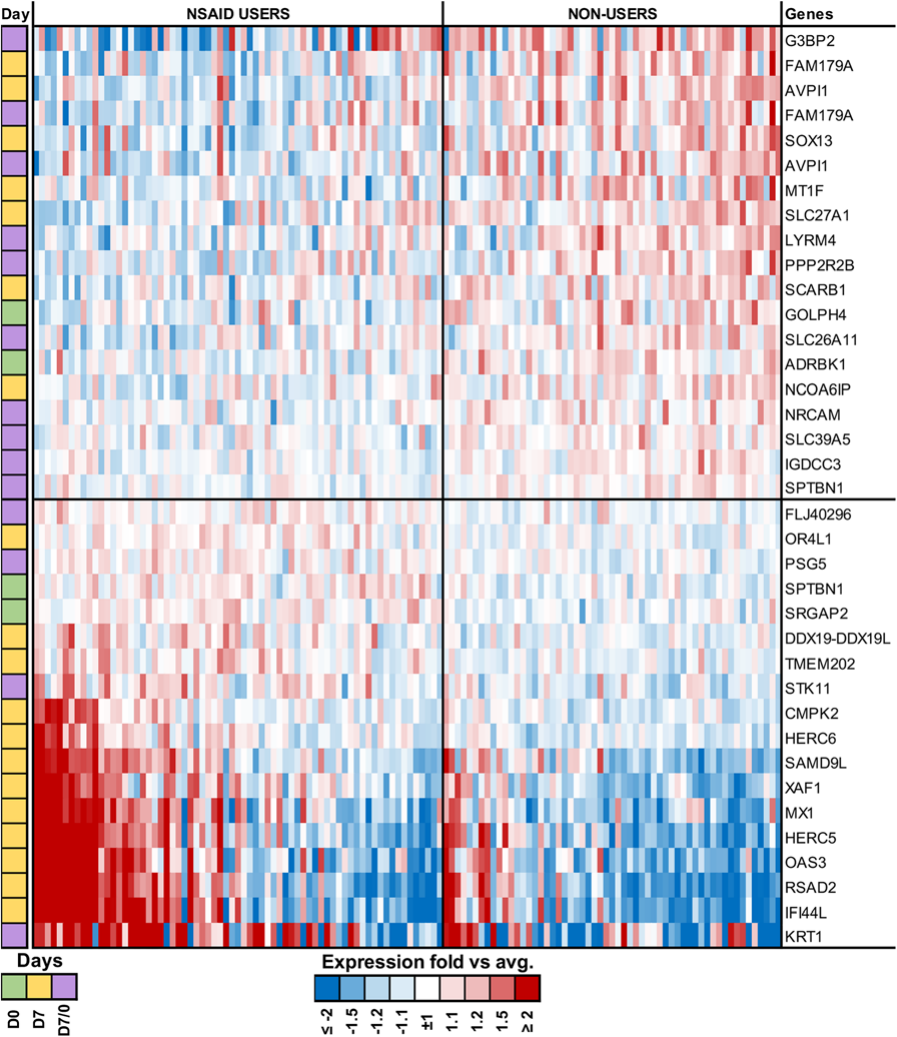
Differences in gene expression profiles in NAIDS users. Gene expression heatmap of known DE genes between individuals on NAIDS or non-users with a False Discovery Rate (FDR) < 10% are displayed. A cut-off of 10% was chosen a priori based on the balance between Type 1 and Type 2 errors as explained further in the methods. The heat-map shows genes that were DE at baseline (D0, day 0, visit 1), 1 week after vaccination (D7, day 7, visit 2) and in response between the two visits (D7/D0). The color in the first column indicates the time point at which the genes were found to be DE between the two groups, whereas the heatmap colors highlight the fold-change. The heatmap is divided into four grids, wherein the bottom left group demonstrates the genes that were significantly upregulated in the NSAIDs-users, and the top right group showcases genes found to be upregulated in the non-users

In the elderly taking NSAIDs, gene expression analysis showed significant differences for 42 probes (FDR < 10%) (Fig. 4). Notably, DAVID identified many of those genes as being involved in antiviral defense, mainly by inhibiting viral genome replication, and mediating innate immunity and the type-1 interferon signaling pathway (Table 1). For example, the gene RSAD2, which encodes Viperin, showed a 1.6-fold increase in NSAIDs users. Viperin disrupts the lipid rafts on cell plasma membranes and inhibits influenza viral budding and release [26]. Its upregulation in NSAIDs users, who were found to have more pronounced Ab production and better VNA titers against H1N1, suggests a possible mechanism by which NSAIDs could alter immune response to the influenza vaccine. Similarly, 39 genes were found to have statistically significant differential expression (FDR < 10%) in the cohort using Statins when compared with non-users (see Additional file 5: Figure S5). Several of these genes encode for proteins that are known to affect major immune-regulatory pathways, such as the OX40 signaling pathway, B cell development, and CD28 and iCOS-iCOSL Signaling in T Helper cells (Table 1).

### Differences in the proportion of immune cells in the drug-using cohorts

**Fig. 5:**
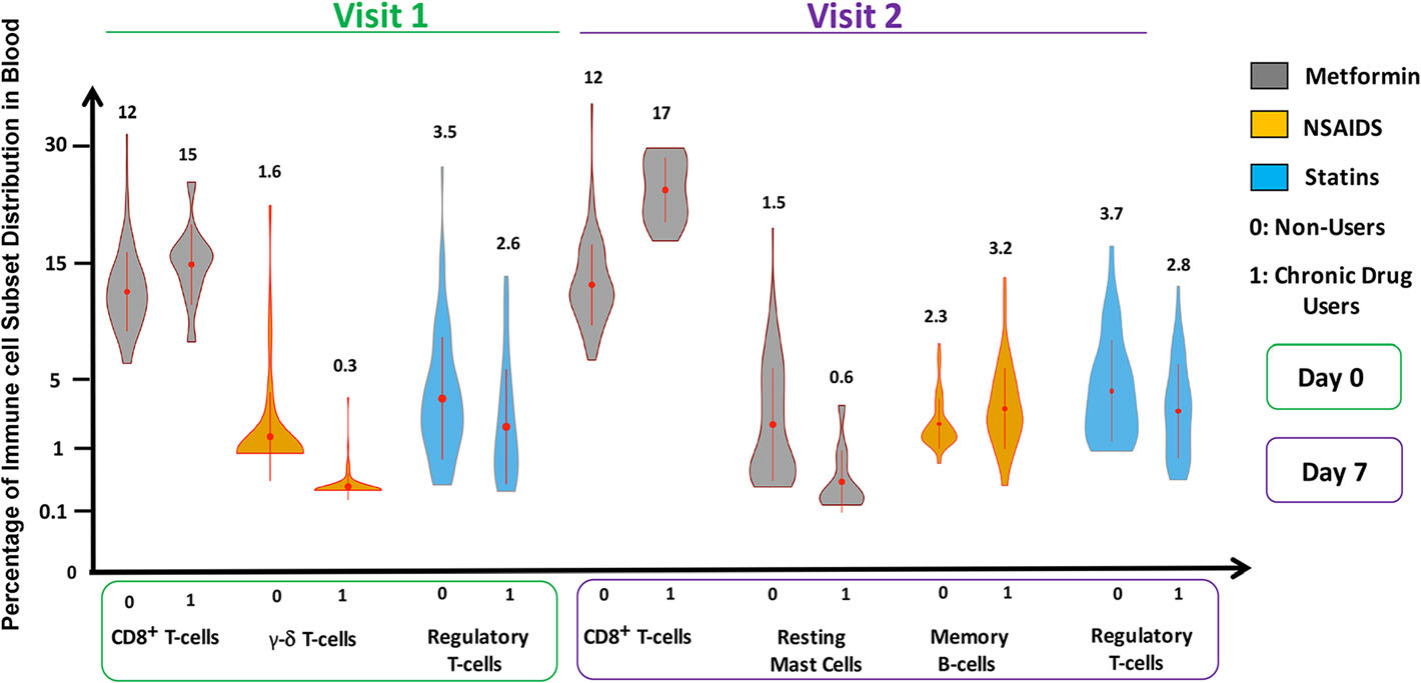
Distribution of circulating lymphocyte subsets. Violin Plots show distribution of different cells types in individuals with a history of Metformin, NSAIDS or Statin use (1 on the X-axis) compared with individuals who have no history of use of these prescription drugs (0 on the X-axis). The red dots in the middle of the plot show median results, which are also shown by numbers on top of the plots. Red lines indicate interquartile ranges. Pair-wise differences between the cohorts were performed using the non-parametric Mann-Whitney U Test. Significant differences between the two cohorts are indicated by stars above the plots. * *p*-values were as follows: Metformin users vs. non-users: CD8^+^ T-cells at baseline: *p*-value = 0.04; CD8^+^ T-cells at 1 week after TIV: *p*-value = 0.01. NSAIDs users compared to non-users: memory B-cells at 1 week after TIV: *p*-value = 0.0004); γ-δ T-cells at baseline (*p*-value = 0.03). Statin users vs. non-users: Regulatory T-cells at baseline: *p*-value = 0.02; regulatory T-cells at 1 week after TIV: *p*-value = 0.04

We assessed cell distribution by deconvoluting the gene expression data using CIBERSORT [27]. The most remarkable changes were in the proportion of CD8^+^ T-cells in individuals using Metformin. At baseline, we found that individuals with a history of long-term Metformin use tend have a 25% higher proportion of CD8^+^ T-cells. One week after influenza vaccination, there appeared to be minimal changes in the proportion of CD8^+^ T-cells in non-users, whereas metformin users had a 41% higher proportion (Fig. 5). Additionally, at visit 2, Metformin users were found to have a 60% lower proportion of resting mast cells. Moreover, when we examined the cell type proportions in individuals with a history of NSAIDs use versus those who were non-users, we found the former group to have a significantly lower quantity of γ-δ T-cells. At visit 2, although this difference was no longer statistically significant, the NSAID group was now found to have a 39% greater fraction of memory B-cells compared to the non-NSAIDs users. We also noted individuals with a history of long-term use of Statins tend to have significantly lower proportions T_reg_ cells, both at baseline and at visit 2 after vaccination. At visit 1, Statin users had on average 25% lower proportion of T_reg_ cells. Although at visit 2, both groups saw an absolute increase of 0.2% in the T_reg_ proportion, Statin users continued to have a 25% lower fraction of T_reg_ cells (Fig. 5).

## Discussion

The current study systematically examined the effects of three commonly used medications on the immune system’s response of the aged to influenza vaccination. We corroborated the differences observed in VNA titer or Ab levels with focused B-cell marker studies as well as global blood transcriptomic analyses. The drugs we analyzed included Metformin, NSAIDs and Statins. Metformin activates AMPK and inhibits mTOR and STAT3 activation [28]. It also activates IKKα/β, reduces ROS production and blunts the secretion of pro-inflammatory cytokines [29]. Metformin increases oxidative phosphorylation and fatty acid catabolism and thereby promotes memory formation of T cells instead of their differentiation into effector cells, which preferentially use glycolysis for energy and biomass production [30, 31]. B cells also switch to glycolysis upon activation and drugs that promote pathways of energy production other than glycolysis might reduce B cell proliferation [32]. Metformin similar to DNA breaks, which occur during immunoglobulin class switch reactions, inactivates the CREB Regulated Transcription Coactivator 2, which in turn promotes B cell differentiation [33]. It is currently unknown how Metformin affects different steps of plasma cell developments; the drug’s effects on glycolysis may dampen while its role on transcriptional regulation may promote antibody responses.

NSAIDs function through inhibition of prostaglandin synthesis and cyclooxygenases 1 and 2 (Cox-1 and Cox-2), two components of the respiratory chain that catalyzes the reduction of oxygen to water [34]. Cox-2 is upregulated in activated human B lymphocytes and is required for optimal Ab synthesis. It has been found that NSAIDs blunt antibody production in stimulated human peripheral blood mononuclear cells (PBMCs) [35] by reducing the expression of Blimp-1 a transcription factor that is crucial for plasma cell differentiation [36]. It also suppresses T-cell activation by inhibiting p38 MAPK induction.

Statins inhibit HMG-CoA reductase and thereby the mevalonate pathway, which leads to production of cholesterol and heme; the latter plays a role in B-cell differentiation and class switching [37]. Statins in part through reduction of intracellular cholesterol, which is an essential cell wall component needed to for dividing cells, reduces cell proliferation [38]. It inhibits cytokine induced increases in MHC class II expression [39] and thereby affects antigen-driven stimulation of CD4^+^ T cells. Statins also impede the production of coenzyme Q10, an electron carrier in the electron transport chain, which shifts the balance from mitochondrial energy production towards glycolysis. They have been shown to decrease CD40/CD40L expression, inhibit T-cell activation as well as diminish Ab production [40].

Previous studies have found correlations between drug use and dysregulation of the immune system. For instance, Metformin use has been shown to promote fatty acid metabolism and increase CD8^+^ memory T-cell formation, thus supporting development of immunological memory [41]. In addition to having lower baseline VNA titers to the H1N1 and H3N2 influenza virus strains, we found that individuals on Metformin also had suppressed Ab responses following vaccination combined with lower expression of metabolic phenotypic markers, such as Sirt1 on Ab secreting and non-switched memory B-cells. Metformin may thus have inhibited formation of ASCs by reducing the metabolic switch to glycolysis, which is a hallmark of lymphocytes undergoing activation and which is essential to provide rapid energy and biomass for proliferating cells. Moreover, we find compelling evidence that both at baseline and a week after vaccination, Metformin users exhibited a significantly higher proportion CD8^+^ T-cells. The majority of the genes involved in the LPS/IL-1–mediated inhibition of RXR function canonical pathway were also downregulated in Metformin users. RXR is a ligand-dependent nuclear receptor that affects transcriptional receptors, and LPS/IL-1 mediated inhibition of RXR function impairs metabolism, transport and biosynthesis of lipids and cholesterol [42], which are essential for membrane formation by proliferating cells. Thus, our results are consistent with previous associations between Metformin’s effects on immunity through metabolic dysregulation. Still yet, these observations propose an overarching mechanism explaining how Metformin potentially inhibits RXR, alters the utilization of fatty acids by lymphocytes and weakens their ability to proliferate. It should be pointed out though that our results of reduced antibody responses to influenza vaccination in Metformin users are in contrast to a previous study, which showed that in individuals with T2D Metformin increases responses [14]. We assume that the contrasting results reflect that in this study patients were younger than our cohorts and Metformin users were compared to diabetic individuals, who had not yet initiated treatment.

Our results show that although absolute VNA titers were higher in individuals receiving NSAIDs, relative to their pre-vaccination baseline there was no statistically significant difference in vaccine responses between users and non-users. This observation is in line with previous studies [43, 44] which have suggested that influenza vaccination effectiveness is likely not reduced in patients taking NSAIDs. In our study, individuals on NSAIDs also had increased levels of BTLA on their naïve mature B-cells, which as we reported previously is linked to higher and more sustained Ab responses to influenza vaccination [7]. One week after vaccination, NSAIDs users had elevated proportion of memory B-cells (Fig. 5). These findings corroborate previous reports and help provide the missing link between NSAIDs, memory B-cells and a possible mechanism by which NSAIDs might exert affect vaccine response in the aged. Recently, it has been found that activated γ-δ T-cells can regulate the organization of B-cells within follicles of lymphoid tissues; they are also crucial in the induction of vaccine-mediated immunity in various animal models [45, 46]. Interestingly, we noted that at baseline, NSAIDs users had a significantly lower proportion of γ-δ T-cells.

Long-term Statin therapy has been associated with a reduced response to the influenza vaccine in elderly individuals [12] and increases in medically attended acute respiratory diseases as well as influenza related office visits or hospitalizations in vaccinated individuals [13, 47]. It has been reported that this is due to its action on the transcription factor Foxp3, which in turn affects differentiation of CD4^+^ T-cells into T_reg_ cells and alters the migration of CD4^+^CD25^+^ T_regs_ [48]. In accordance with previous studies, we found that Statin users have enfeebled IgG and IgM responses against both H1N1 and H3N2 strains of the influenza virus. Although we observed modestly higher baseline VNA titers in Statin users compared with non-users, the rate of titer increase at visits 2 and 3 was comparable between the two cohorts. Higher absolute titers could likely be due to better induction of long-term ASCs in individuals with long-term Statin use. Using deconvolution on microarrays obtained from individuals before vaccination and one week post-vaccination, we demonstrated that Statin users have significantly lower proportion of circulating T_regs_ at both time points. Furthermore, reduced responses were linked to reduced expression of Bcl-6 on both switched memory B-cells and ASCs. Bcl-6 is crucial for germinal cell formation and inhibits terminal differentiation of B-cells into plasma cells. On the other hand, PD-L1, which facilitates interactions between B-cells and follicular T helper cells, was increased on transitional and naïve B-cells as well as ASCs from a Statin user.

The fundamental limitation of a correlative analytic approach like ours is that establishment of perfect causal order is not always possible. Our findings are thus not meant to settle the issues of drug-vaccination interaction in the elderly, but rather contribute to a dialogue that has only recently commenced. These observations are all the more stimulating because the three drugs under investigation in this study are widely and commonly used by the aged, who are also the most vulnerable population to the complications of an influenza infection. Other limitations of our study include its modest sample size, which might explain why many of the Ab and VNA titer differences observed between Statin/NSAIDs users and non-users did not reach statistical significance. Due to the limited number of human subjects enrolled in the study, we also did not consider drug-drug interaction because less than 10% of the individuals were on multiple drugs, and such a sample size would drastically limit the power to detect any meaningful differences as well as limit our confidence in the biological validity of the results. Additionally, most of the individuals in the study were Caucasian women because of geographical and patient recruitment reasons. Sex and race differences in immunity have been well-documented, and in general, women have been shown to develop higher Ab responses than males [3], although we failed to confirm this trend in our studies. Other potential limitations are that additional factors, which could potentially influence immune responses, such as overall fitness, levels of daily physical activity, underlying chronic diseases, use of other prescription drugs, diet and weight, and others, were not taken into account. Thus, caution must be taken when interpreting our results across individuals from different ethnic/racial backgrounds, and we encourage future research to look at the epistasis between a patients’ history of medication use and other factors such as sex and gender. Since we followed 67 individuals over 5 years and used their data to evaluate four conditions. i.e., Metformin use, NSAIDs use, Statin use and no medication use, our conclusions are affected by both Type 1 and Type 2 errors. Nonetheless, our study addresses a fundamental gap in knowledge regarding a clinically relevant topic of how chronic drug use affects vaccine response in the aged, and opens the door for future studies to replicate our preliminary findings in larger, diverse cohorts.

## Conclusions

We show that compared with non-users, Metformin users exhibit significantly lower increases in VNA titers and Abs tested for by an ELISA after influenza vaccination, while individuals using NSAIDs and Statins developed absolute higher responses but as their baseline titers tended to be higher, their overall increases in influenza virus-specific Abs following vaccination were also attenuated. We correlated these observations with differences in B cell phenotypes, whole transcriptome analyses and inferred immune cell distribution based on deconvolution. Subsequent pathway analysis reveals mechanistic routes underlying the medication-immunity interaction in response to vaccination. We conclude that chronic medication use in the aged population significantly impacts immune responses to vaccination, and greater emphasis must be placed on medication history while considering alternate protective strategies for the elderly to reduce influenza-associated mortality. Overall, our results are important in two ways: first, they offer an important step in the ongoing effort to explain the differential responses to the influenza vaccine in the exposed elderly population. Second, they provide a partial road map to vaccine researchers and immunologists who can begin the transition from documenting the effects of long term medication use on immunosenescence to explaining how medication use can be exploited or countered to ensure that the elderly are adequately protected by the influenza vaccine. In summary, we generate novel data to explain how chronic medication use in the elderly affects their immune system, and in turn alter their responsiveness to vaccination.

## Methods

### Human subjects and study design

Blood was collected in the Duke Clinical Research Unit (DCRU) after informed consent from community dwelling persons in the Durham-Raleigh-Chapel Hill area of North Carolina (USA). Eighty healthy individuals, ≥ 65 years of age, who did not meet the criteria for age-related frailty, were included in the study. Subjects with acute febrile infections or underlying diseases or therapies that affect the immune system were not enrolled. Subjects with contraindication to the influenza vaccination, such as anaphylactic hypersensitivity to eggs or to other components of the vaccine, or acute illness with or without fever, or a history of Guillain-Barré Syndrome within 6 weeks following a previous dose of influenza vaccine were not enrolled. Demographic data and medical history including medical diagnoses, medications, past vaccination, and history of influenza or influenza-like diseases during the last 5 years were recorded. Subjects were bled and vaccinated with TIV. Subjects were bled again on days 7 and 14 or 28, following the injection of TIV.

### Virus strains

The egg-adapted influenza A vaccine strains, H1N1 and H3N2, present in the vaccines during our study were obtained from the Center for Disease Control and Prevention (CDC), Atlanta, Georgia. The same H1N1 strain i.e., A/California/7/2009, was used throughout the entire period. The H3N2 stain was changed throughout the study period: 2011/12: A/Perth/16/2009, 2–12/2013/2014: A/Victoria/361/2011; 2014/15: A/Texas/50/2012; 2015/16: A/Switzerland/9715293/2013. Viruses were expanded in 10-day-old, pathogen-free embryonated eggs. Cleared allantoic fluids were purified by fractionation over 10–55% sucrose density gradients at 25,000 rpm for 2 h. Mean tissue culture infective doses (TCID_50_) were determined by titration on Madin-Darby Canine Kidney (MDCK) cells after 3 days of infection by screening for cytopathic effects (CPE).

### Blood and serum samples

Blood was collected from the enrolled individuals. The blood samples for the gene expression analyses were collected in PaxGene tubes to immediately stabilize RNA for analysis as described previously [49]. Samples were shipped overnight from the point of collection in the DCRU to The Wistar Institute in Philadelphia, PA (USA). Sera were isolated and frozen at − 20 °C until further use. Sera were heat-inactivated by a 30-min incubation at 56 °C prior to testing.

### Micro-neutralization assay

Two-fold serially diluted (1:20 to 1:10240) heat-inactivated human sera were tested for neutralizing Abs to influenza A virus strains by micro-neutralization assays [50]. Briefly, equal volume of 100 TCID_50_ per well of the titrated virus was added to the diluted serum in 96-well plates and incubated at 37 °C. After 1 h, serum-virus mixtures were added to MDCK cells that had been washed twice with serum-free Dulbecco’s Modified Eagles Medium (DMEM). The cells were incubated for two hours at 37 °C with 5% CO_2_. The cells were washed and re-incubated with DMEM supplemented with L − 1-Tosylamide-2-phenylethyl chloromethyl ketone (TPCK) trypsin for 3 days. CPEs were scored under a microscope. Neutralization titers were defined as the dilution of the serum that resulted in 50% inhibition of CPE formation.

### Enzyme-linked immunosorbent assay (ELISA)

H1N1- and H3N2-specific binding Ab isotypes were measured by ELISA. Briefly, wells of Nunc Maxisorp™ plate were coated with 10 μg/ml of influenza H1N1 or H3N2 virus along with isotype standards for IgA1, IgG and IgM (Athens Research & Technology, Inc., Georgia, USA) in bicarbonate buffer overnight at 4 °C. The plates were blocked with 3% BSA in phosphate-buffered saline (PBS) and incubated for 2 h. at room temperature with heat-inactivated sera of subjects at a dilution of 1/250. The plates were washed 4X with PBS containing 0.05% PBS-tween (PBST) and incubated for 1 h. at room temperature with alkaline phosphatase conjugated mouse anti-human IgA1 at 1:1000, IgG at 1:3000 and IgM at 1:1000 dilutions (Southern Biotech, Alabama, USA). Following the incubation, plates were washed 4X with PBST and developed using alkaline phosphatase substrate containing pNPP tablets (Sigma Aldrich, Missouri, USA) dissolved in DEA buffer. Adsorbance values were recorded at 405 nm and plotted against standard curves from each plate for every isotype. Ab concentrations were determined, and expressed in μg/ml.

### B cell detection by flow cytometry

Peripheral blood mononuclear cells (PBMCs) were isolated as previously described [7]. Each subject’s PBMC sample was initially treated with Human TruStain FcX Fc Receptor Blocking solution (BioLegend, San Diego, CA) for 30 min, washed with PBS at 1500 rpm for 5 min and then stained with fluorochrome-conjugated Abs. Multi-parametric flow cytometry was performed on the PBMCs using a panel to detect B cell subsets by identifying mature naïve B cells (CD19^+^CD20^+^IgD^+^CD27^−^CD38^−^), transitional B cells (CD19^+^CD20^+^IgD^+^CD27^+/-^CD38^+/−^), non-switched memory B cells (CD19^+^CD20^+^IgD^+^CD27^+^CD38^−^), switched memory B cells (CD19^+^CD20^+/-^IgD^−^CD27^+^CD38^−^), double-negative B cells (CD19^+^CD20^+^IgD^−^CD27^−^CD38^−^) and ASCs (CD19^+^CD20^−^IgD^−^CD27^++^CD38^++^). PBMCs were stained with Ab conjugates for the following surface markers: CD3-Pacific Blue (UCHT1, Biolegend, San Diego, CA), CD14-Pacific Blue (M5E2, Biolegend), CD19-BV650 (HIB19, Biolegend), CD20-BV570 (2H7, Biolegend), CD27-BV785 (O323, Biolegend), CD38-BV711 (HIT2, Biolegend), IgD-PerCP/Cy5.5 (IA6–2, Biolegend), BTLA-PE (MIH26, Biolegend), PD1-PE/Cy7 (EH12.2H7, Biolegend), and Live/Dead Fixable Aqua Dead Cell Stain (Life Technologies, Carlsbad, CA) for 30 min at 4 °C.

Cells were washed twice with cell staining buffer (Biolegend), and fixed using Cytofix/Cytoperm (BD Biosciences). Intracellular antigens were detected by staining for IgG-BV605 (G18–145, BD Biosciences, San Jose, CA) and IgM-APC/Cy7 (MHM-88, Biolegend) for 30 min at 4 °C. The stained samples were analyzed in a LSRII flow cytometer (BD Biosciences, San Jose, CA) using FlowJo (Tree Star, Ashland, OR).

### Microarray data analysis and statistical inference

PAXgene tubes were stored at − 80 °C until RNA extraction. RNA was extracted using the PAXgene Blood RNA Kit IVD for isolation and purification of intracellular RNA according to the manufacturer’s directions. RNA integrity (RIN) was assessed using a bioanalyzer and only samples with a RIN score of > 7.5 were processed for arrays. A constant amount (400 ng) of total RNA was amplified, as recommended by Illumina, and hybridized to the Illumina H12-v4 human whole genome bead arrays. All arrays were processed in the Wistar Institute Genomics Facility. Illumina GenomeStudio software was used to export expression levels and detection *p*-values for each probe of each sample. From individual hybridizations, data normalization and transformation to make meaningful comparisons of expression levels and select genes for further analysis and data mining was done as previously described [51]. Signal intensity data was quantile normalized and log2 transformed. Expression level comparisons between two groups were done using two-sample t-test and the Benjamini-Hochberg (BH) correction for multiple testing was performed [52]. A False Discovery Rate (FDR) < 10% was used as a significance threshold. We decided the cut-off of 10% a priori based on a balance of tolerance for Type 1 vs. Type 2 errors. An FDR of 0.1 ensures that majority of the genes detected as differentially expressed likely represent true biological differences, yet it provides us with an optimum tolerance of false positives to maximize the discovery of real biological signals.

Additionally, we used the gene-expression data to estimate the abundances of different immune cell types in the mixed blood cell population. The deconvolution was performed using CIBERSORT, a support vector regression based model [27]. We then compared the differences in cell type composition in the samples collected from individuals who were on a given medication versus those who were not, using the Mann-Whitney U Test. Gene set enrichment analysis for biological functions and canonical pathways was also performed using the Database for Annotation, Visualization and Integrated Discovery (DAVID) *v*6.8 [53]. All statistical analyses were conduction using the statistical language *R v*3.4.3*.* Results with BH corrected *p*-values < 0.05 were considered significant unless noted otherwise.

## Additional files

Additional file 1: **Figure S1.** Serum samples collected on two separate visits following vaccination were tested for IgA, IgG and IgM to H1N1 and H3N2 viruses by ELISA. The violin plots as described in legend to Fig. 2 show the median Ab levels between the two groups. Pair-wise differences between the two cohorts in each of the six panels were performed using the Mann-Whitney U Test, * *p*-values ≤0.05 and ** *p*-values ≤0.005. (PDF 728 kb)

Additional file 2: **Figure S2.** The boxplots show MFI levels of significantly altered markers, PD1 and BTLA, on different B-cell subsets from individuals with no history of medication use, compared with individuals using Metformin, NSAIDs or Statins as described in legend to Fig. 3. The B-cell subsets were identified using flow cytometry. Pair-wise differences between the cohorts in each panel were performed using the Mann-Whitney U Test. Significant differences are indicted by stars: * *p*-values ≤0.05, ** *p*-values ≤5 × 10^− 3^, *p*-value ≤5 × 10^− 4^. (PDF 876 kb)

Additional file 3: **Figure S3.** The boxplots show MFI levels of significantly altered markers, Bcl-6, MROS and PD-L2, on different B-cell subsets from individuals with no history of medication use, compared with individuals using NSAIDs. as described in legend to Fig. 3. Pair-wise differences between the cohorts in each panel were performed using the Mann-Whitney U Test, Significant differences are indicted by stars: * *p*-value ≤0.05, ** *p*-value ≤5 × 10^− 3^, *** ^p^*-*value ≤5 × 10^− 4^. (PDF 866 kb)

Additional file 4: **Figure S4.** Differentially expressed (DE) genes in the chronic Metformin-user cohort compared with non-users. Gene expression heatmap of known DE genes between the two cohorts with a False Discovery Rate (FDR) of < 10% are displayed. The heat-map indicates genes that were DE at baseline (D0, day 0, visit 1), 1 week after vaccination (D7, day 7, visit 2) and in response between the two visits (D7/D0). (PDF 5059 kb)

Additional file 5: **Figure S5.** Differentially expressed (DE) genes in the chronic Statin-user cohort compared with non-users. Gene expression heatmap of known DE genes between the two cohorts with a False Discovery Rate (FDR) of < 10% are displayed. The heat-map indicates genes that were DE at baseline (D0, day 0, visit 1), 1 week after vaccination (D7, day 7, visit 2) and in response between the two visits (D7/D0). (PDF 4428 kb)

## Abbreviations

Ab: Antibody
ASCs: Ab secreting cells
Bcl6: B-cell lymphoma 6
BH: Benjamini-Hochberg
BTLA: B and T lymphocyte attenuator
CD: Cluster of Differentiation
CDC: Centers for Disease Control and Prevention
Cox: Cyclooxygenase
CPE: Cytopathic effects
DAVID: Database for Annotation, Visualization and Integrated Discovery
DCRU: Duke Clinical Research Unit
DMEM: Dulbecco’s Modified Eagles Medium
ELISA: Enzyme-linked immunosorbent assay
Ig: Immunoglobulin
MDCK: Madin-Darby Canine Kidney
MFI: Mean Florescence Intensity
MROS: Mitochondrial Reactive Oxygen Species
NSAIDs: Non-steroidal anti-inflammatory drugs
Oct2: Octamer transcription factor 2
PBMC: Peripheral blood mononuclear cell
PBS: Phosphate Buffered saline
PD1: Programmed cell death protein 1
PD-L: Programmed death-ligand
RIN: RNA integrity
ROS: Reactive Oxygen Species
Sirt1: Sirtuin 1
TCID: Tissue culture infective dose
TIV: Trivalent inactivated influenza vaccine
TPCK: Tosylamide-2-phenylethyl chloromethyl ketone
Treg: Regulatory T-cells
VNA: Virus-neutralizing Ab
