# Supplementary material for: Immune response to influenza vaccination in the elderly is altered by chronic medication use
Source: Immun Ageing. 2018 Aug 31;15:19. doi: 10.1186/s12979-018-0124-9 (PMC6119322; doi:10.1186/s12979-018-0124-9)
Supplement: Supplementary file 1 — Figure S1. Serum samples collected on two separate visits following vaccination were tested for IgA, IgG and IgM to H1N1 and H3N2 viruses by ELISA. The violin plots as described in legend to Fig. 2 show the median Ab levels between the two groups. Pair-wise differences between the two cohorts in each of the six panels were performed using the Mann-Whitney U Test, * p-values ≤0.05 and ** p-values ≤0.005. (PDF 728 kb) [file 12979_2018_124_MOESM1_ESM.pdf]

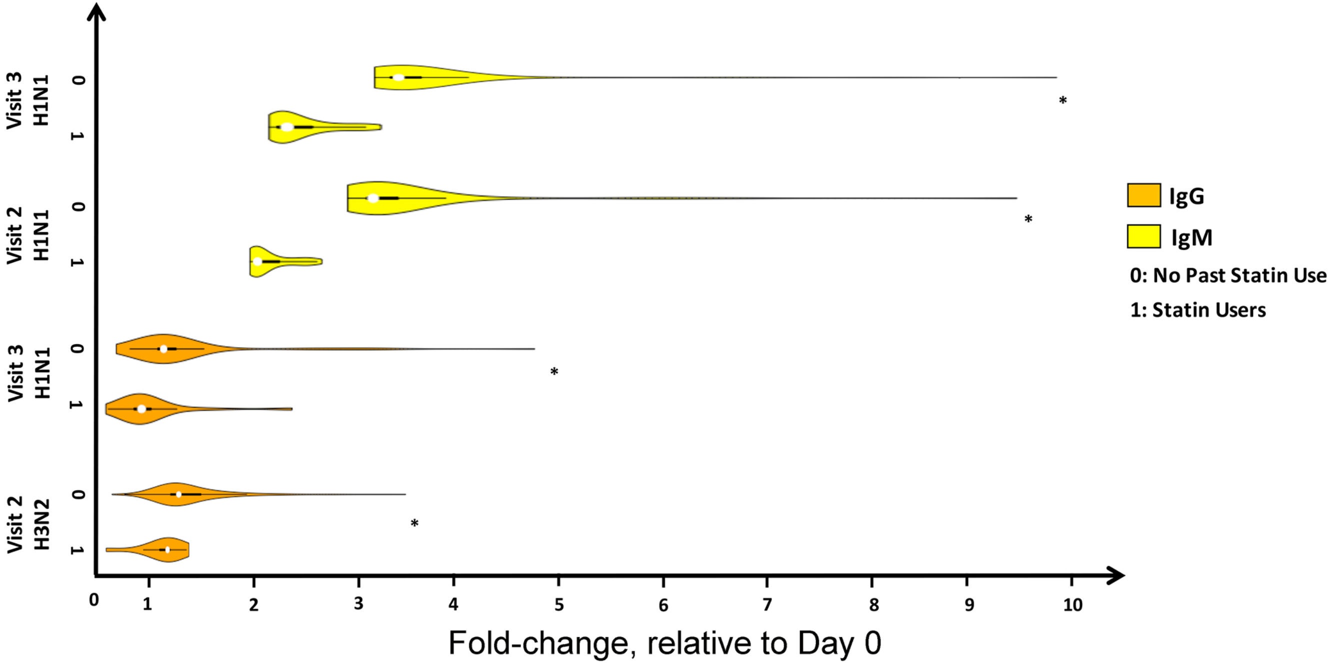

Supplementary Fig. 1

Serum samples collected on two separate visits following vaccination were tested for IgA, IgG and IgM to H1N1 and H3N2 viruses by ELISA. The violin plots as described in legend to Figure 2 show the median Ab levels between the two groups. Pair-wise differences between the two cohorts in each of the six panels were performed using the Mann-Whitney U Test, * p-values ≤ 0.05 and ** p-values ≤ 0.005.
