# Supplementary material for: Immune response to influenza vaccination in the elderly is altered by chronic medication use
Source: Immun Ageing. 2018 Aug 31;15:19. doi: 10.1186/s12979-018-0124-9 (PMC6119322; doi:10.1186/s12979-018-0124-9)
Supplement: Supplementary file 2 — Figure S2. The boxplots show MFI levels of significantly altered markers, PD1 and BTLA, on different B-cell subsets from individuals with no history of medication use, compared with individuals using Metformin, NSAIDs or Statins as described in legend to Fig. 3. The B-cell subsets were identified using flow cytometry. Pair-wise differences between the cohorts in each panel were performed using the Mann-Whitney U Test. Significant differences are indicted by stars: * p-values ≤0.05, ** p-values ≤5 × 10− 3, p-value ≤5 × 10− 4. (PDF 876 kb) [file 12979_2018_124_MOESM2_ESM.pdf]

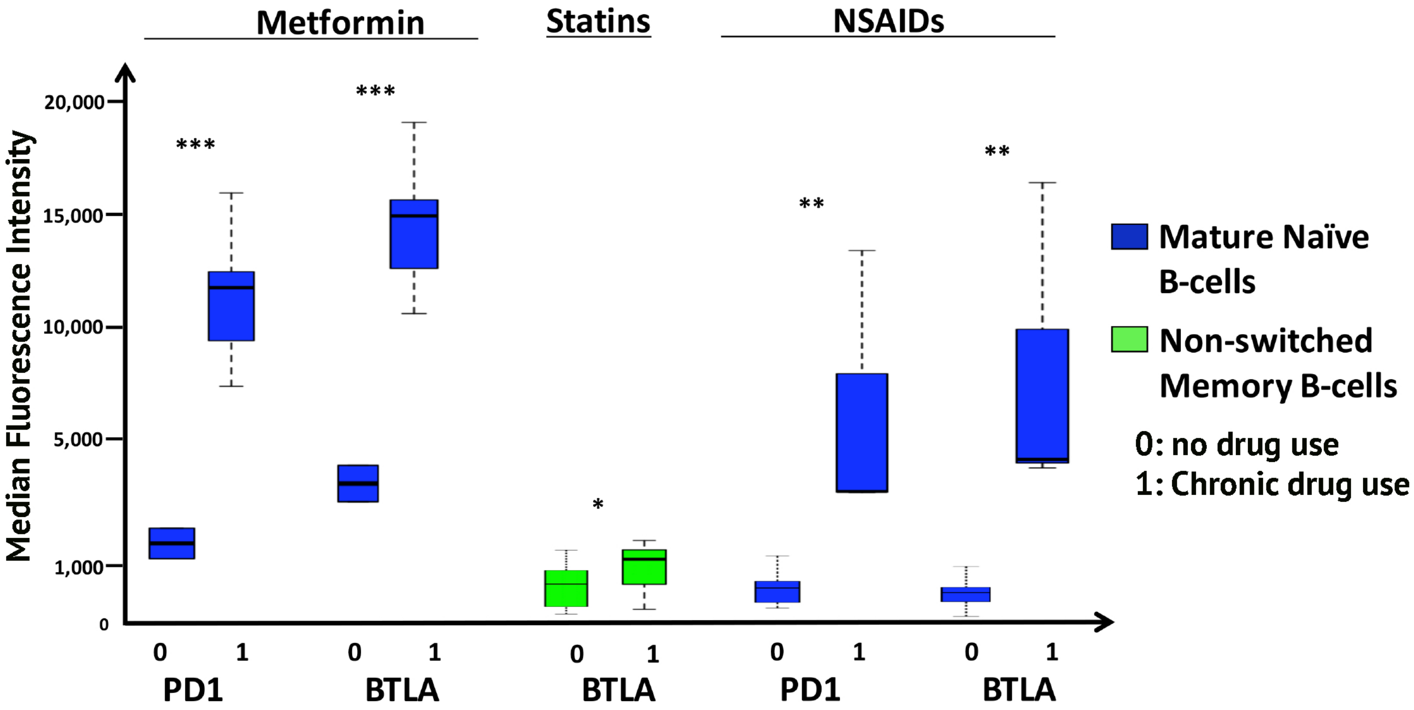

Supplementary Fig. 2

The boxplots show MFI levels of significantly altered markers, PD1 and BTLA, on different B-cell subsets from individuals with no history of medication use, compared with individuals using Metformin, NSAIDs or Statins as described in legend to Figure 3. The B-cell subsets were identified using flow cytometry. Pair-wise differences between the cohorts in each panel were performed using the Mann-Whitney U Test. Significant differences are indicted by stars: * p-values ≤ 0.05, ** p-values ≤ $5x10^{-3}$, p-value ≤ $5x10^{-4}$.
