# Supplementary material for: Immune response to influenza vaccination in the elderly is altered by chronic medication use
Source: Immun Ageing. 2018 Aug 31;15:19. doi: 10.1186/s12979-018-0124-9 (PMC6119322; doi:10.1186/s12979-018-0124-9)
Supplement: Supplementary file 4 — Figure S4. Differentially expressed (DE) genes in the chronic Metformin-user cohort compared with non-users. Gene expression heatmap of known DE genes between the two cohorts with a False Discovery Rate (FDR) of < 10% are displayed. The heat-map indicates genes that were DE at baseline (D0, day 0, visit 1), 1 week after vaccination (D7, day 7, visit 2) and in response between the two visits (D7/D0). (PDF 5059 kb) [file 12979_2018_124_MOESM4_ESM.pdf]

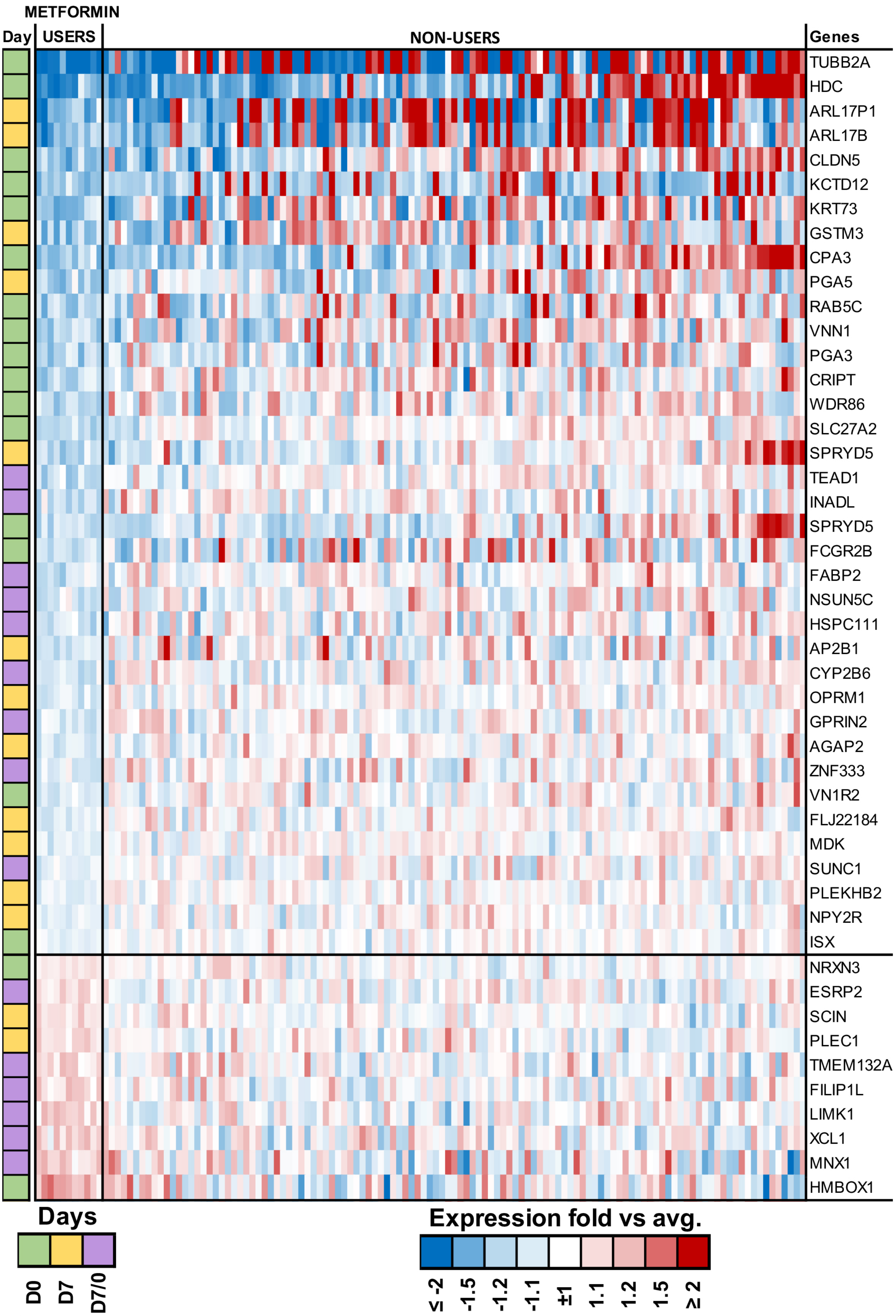

Supplementary Fig. 4

Differentially expressed (DE) genes in the chronic Metformin-user cohort compared with non-users. Gene expression heatmap of known DE genes between the two cohorts with a False Discovery Rate (FDR) of <10% are displayed. The heat-map indicates genes that were DE at baseline (D0, day 0, visit 1), 1 week after vaccination (D7, day 7, visit 2) and in response between the two visits (D7/D0).
